# Analysis of Emergency Department Visits and Hospital Activity during Influenza Season, COVID-19 Epidemic, and Lockdown Periods in View of Managing a Future Disaster Risk: A Multicenter Observational Study

**DOI:** 10.3390/ijerph17228302

**Published:** 2020-11-10

**Authors:** Enrique Casalino, Christophe Choquet, Donia Bouzid, Olivier Peyrony, Sonja Curac, Eric Revue, Jean-Paul Fontaine, Patrick Plaisance, Anthony Chauvin, Daniel Aiham Ghazali

**Affiliations:** 1 Emergency Department, Assistance Publique-Hôpitaux de Paris, Hôpital Bichat, 75018 Paris, France; enrique.casalino@aphp.fr (E.C.); christophe.choquet@aphp.fr (C.C.); donia.bouzid@aphp.fr (D.B.); 2 Study Group for Efficiency and Quality of Emergency Departments and Non-Scheduled Activities Departments, Assistance Publique-Hôpitaux de Paris, 75018 Paris, France; olivier.peyrony@aphp.fr (O.P.); sonja.curac@aphp.fr (S.C.); eric.revue@aphp.fr (E.R.); jean-paul.fontaine@aphp.fr (J.-P.F.); patrick.plaisance@aphp.fr (P.P.); anthony.chauvin@aphp.fr (A.C.); 3 IAME (Infection, Antimicrobial, Modeling, Evaluation), INSERM UMR1137, Université de Paris, 75018 Paris, France; 4 Emergency Department, Assistance Publique-Hôpitaux de Paris, Hôpital Saint Louis, 75010 Paris, France; 5 Emergency Department, Assistance Publique-Hôpitaux de Paris, Hôpital Beaujon, 92110 Clichy, France; 6 Emergency Department, Assistance Publique-Hôpitaux de Paris, Hôpital Lariboisière, 75010 Paris, France; 7 Centre of Research in Epidemiology and Statistics, INSERM UMR1153, Université Sorbonne, 75004 Paris, France; 8 Emergency Medical Services, Assistance Publique-Hôpitaux de Paris, Hôpital Beaujon, 92110 Clichy, France

**Keywords:** COVID-19, seasonal influenza, time series analysis, lockdown, emergency department activity, hospital admissions

## Abstract

ED-visits and through-ED admissions to medical/surgical wards (MSW) and intensive care unit (ICU) during influenza, COVID-19 and lockdown periods were evaluated in a four-hospital prospective observational study from November 2018 to March 2020. ED visit characteristics and main diagnostic categories were assessed. Analysis of 368,262 ED-visits highlighted a significantly increasing trend in ED-visits during influenza followed by a significantly decreasing trend after lockdown. For MSW-admissions, a pattern of growth during influenza was followed by a fall that began during COVID-19 pandemic and intensified during the lockdown. For ICU-admissions, a significant rise during the COVID-19 pandemic was followed by diminution during the lockdown period. During lockdown, significantly diminishing trends were shown for all diagnostic categories (between −40.8% and −73.6%), except influenza-like illness/COVID cases (+31.6%), Pulmonary embolism/deep vein thrombosis (+33.5%) and frequent users (+188.0%). The present study confirms an increase in demand during the influenza epidemic and during the initial phase of the COVID-19 epidemic, but a drop in activity during the lockdown, mainly related to non-COVID conditions. Syndromic surveillance of ILI cases in ED is a tool for monitoring influenza and COVID-19, and it can predict ED activity and the need for MSW and ICU beds.

## 1. Introduction

SARS-CoV-2, the virus responsible for COVID-19 disease, was first reported in China and then became a worldwide pandemic [1]. Many countries, including some European countries and the United States, experienced an epidemic peak in late winter/early spring 2020, which compromised the response capacity of their health care systems [2]. Ebola virus disease (EVD) as emerging infectious disease or epidemic in West Africa was more severe than the outbreak itself. It resulted in resource diversion, hospital closures, and led to dramatic reductions in healthcare utilization [3,4,5,6]. A reduction in the number of visits to the emergency department (ED) [7,8,9,10,11,12], orthopedic [13], and urologic emergencies [14] during the COVID-19 epidemic was recently reported. There was also a decrease in hospitalizations from EDs [7], emergency surgery activity [15], and acute coronary syndromes [16].

The epidemic peak of COVID-19 in Europe and particularly in France occurred only a few days after the epidemic peak of seasonal influenza [17]. One of the main drivers of increased winter mortality (observed minus expected, i.e., the deviation from the expected baseline during a season as compared to previous seasons) is seasonal influenza [18]. Seasonal influenza epidemic periods have been associated with an increase in use of EDs and number of hospitalizations in medical/surgical wards (MSW) and intensive care units (ICU) [19,20]. It has been associated with an excess mortality rate of 9.1% in the winter season 2014/2015 as compared to 2014 [21] and in the winter season 2017/2018 [18]. On the other hand, the COVID-19 pandemic was associated with a decrease in ED visits and an increase in hospital admissions [22,23]. Song et al. found that children with influenza in 2019/2020 and COVID-19 in 2020 had similar hospitalization rates [24]. Influenza and COVID-19 were followed by a lockdown strategy to reduce the spread of COVID-19, with numerous political, social, economic, and psychological consequences [2,25]. Following China and some other European countries, by implementing a lockdown from 17 March to 11 May 2020 designed to reduce the spread of COVID-19, France was aiming to curb a dramatic increase of hospitalizations and ICU admissions that was leading to saturation of the health care system [26].

To our knowledge, no study has evaluated the impact of the whole period of seasonal influenza, COVID-19, and lockdown periods on hospital activity including ED visits and through-ED admissions to MSW and ICU. The objective of this multicenter study out of COVID-19 hotspot was to evaluate the trends in hospital activity during this entire period, to compare the activity during the influenza season with lockdown period, and to specify the associated factors with a variation in hospital activity.

## 2. Methods

### 2.1. Study Design and Setting

This is a four-hospital prospective observational non-interventional study. It was conducted from 25 November 2018 to 25 November 2019 (pre-epidemic period) and from 26 November 2019 to 26 March 2020 (study period (influenza- and COVID-19-epidemic and lockdown periods)), all ED visits to four EDs (Bichat, Beaujon, Lariboisière, and Saint-Louis academic hospitals located in the North Paris metropolitan area) and through-ED admissions to MSW and ICU were included.

### 2.2. Definitions and Outcomes

Influenza seasonal epidemic period started on 22 December 2019 [17] and COVID-19 epidemic period on 1 March 2020 [27]. Lockdown was an intervention designed to relieve hospitals under stress due to high patient flow, and introduced on 17 March 2020. Actual COVID-19 cases trend in France is reported on a scatterplot (Appendix A), based on the French government data [28]. All four hospitals are part of an academic hospital trust and have the same computer system for collecting demographic data and coding ED stays. In this trust, all patients (including homeless and poor individuals) have similar access to the ED and rapid diagnostic tests for infectious diseases [29] without any socio-economic disparities. Patients were tested in the ED using a rapid multiplex PCR including SARS-CoV-2 detection [30]. During the pre-study period, data were retrospectively collected. During the study period, daily values for all studied variables were extracted automatically in real time from the electronic medical and hospital database records. The following variables were studied: patient characteristics (age and sex), consultation characteristics including arrival mode (own resources, ambulance, other), triage status, the discharge diagnosis field of the medical ED report, and the discharge procedure from the ED [12]. Triage acuity level is routinely assessed according to the Canadian Triage scale from 1 to 5 (1 = high acuity or complexity, 5 = low acuity or complexity) [31]. We then calculated the number of daily visits, admissions to MSW and to ICU and analyzed by age group (15–50; 50–65; 65–75; ≥75 years) and according to the classification of patients as elderly or not (<65 and ≥65 years). These analyses were also carried out according to triage level categories (1 to 3 and 4 to 5) and to clinical diagnosis. Frequent users were defined as patients with three or more visits during the study period. For each visit to ED, emergency physicians code a diagnosis, which is considered the primary diagnosis. We have selected diagnoses that describe the main situations and reasons for ED visits. As previously reported, influenza-like illness (ILI) includes patients with severe respiratory symptoms, community-acquired pneumonia, fever, acute respiratory illness, and similar septic syndromes [32]. COVID-19 cases include patients with acute symptoms suggestive of COVID-19 infection [33]. An ILI/COVID group was created as some symptoms are common to the two categories. 

### 2.3. Ethics Statement

Data collection and storage by the Urqual^®^ Emergency Database were approved by the French National Commission for Data Protection and Liberties. All data are completely anonymous. The Emergency Ethics Committee for Biomedical Research of Assistance Publique-Hôpitaux de Paris approved this study (project identify code: U-2020-5.3).

### 2.4. Analysis

Interrupted time series (ITS) analysis was used as a statistical comparison of time trends before and after an intervention. ITS at multiple time points before (pre-epidemic period to evaluate seasonality) and after the start of each event (study period: influenza, COVID-19 and lockdown) was conducted to determine whether the different events had a significantly greater effect than any underlying trend, and to analyze the impact of the lockdown period as an intervention aiming to control the influx of patients. ITS currently assesses whether an event or shift in policy was associated with a change in the trend of a target variable. Time series analysis has many applications outside of intervention studies, including economic forecasting, and in the social sciences [34]. The method used included time series analyses (continuous time) and interrupted time series (ITS) [35,36]. Autoregressive integrated moving average (ARIMA) models, using Box–Jenkins methodology, were used to evaluate seasonal effects, i.e., whether relationships existed between the studied groups and the different epidemic and lockdown periods [35,37]. The model was identified by determining the ARIMA model using autocorrelation function and Partial Autocorrelation function, which are necessary to identify the right ARIMA model parameters, p, autoregression order; d, difference order; q, moving average order, as well as Seasonal Autoregressive Integrated Moving Average, which is an extension of the ARIMA model to evaluate seasonal effects. We went on to evaluate: P, seasonal autoregressive order; D, seasonal difference order; Q, seasonal moving average order; m, seasonal period (annual period 12, quarter 4). The adequacy of the model was checked, and the statistical significance of the parameters was determined. Omega estimates the difference between the data series before and after the intervention; delta indicates the duration of any change and the rate of recovery if present. Omega indicates the overall shift; the sign represents the direction of the change, and delta represents the speed at which a gradual increase or decrease of these initial changes occurred over time. Both parameters should be statistically significant so as not to end up with paradoxical conclusions. We then built a multifaceted analysis model based on daily visits, MSW and ICU admissions, and demographic and ED stay characteristics of interest. We selected the relevant variables based on the adjusted R^2^, with a stepwise approach. Trends in the numbers of studied variables were obtained as daily values were evaluated by linear regression, with days as the sole explanatory variable.

Statistica v12 software (Statsoft, Tulsa, OK, USA) was used for analysis. We deemed statistical differences to be significant for *p* < 0.05. 

## 3. Results

**Figure 1:**
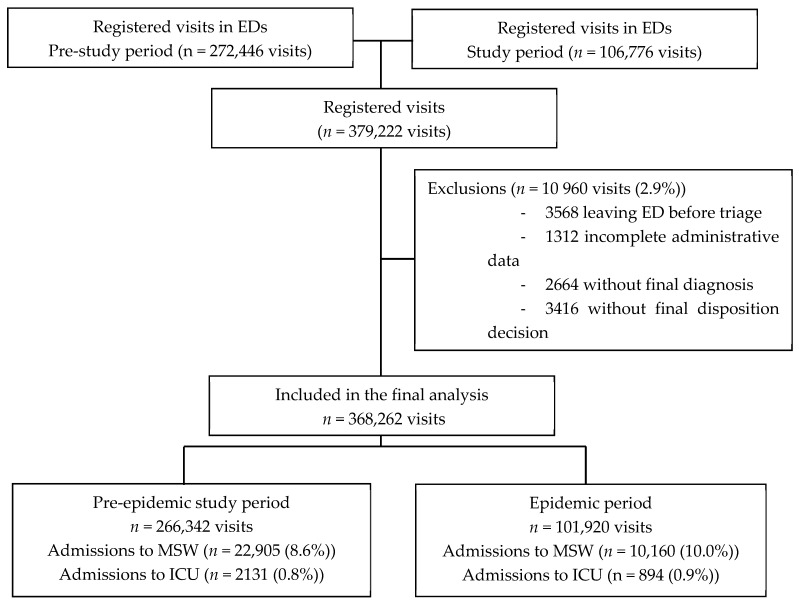
Flow chart. Legend: ED, Emergency department; ICU, intensive care unit; MSW, medical/surgical wards.

During the study period, 379,222 ED visits were registered, and 368,262 were finally analyzed (97.1%). Figure 1 presents the study flowchart. 

### 3.1. Trends in ED Visits and Through-ED Admissions to MSW and ICU

**Figure 2:**
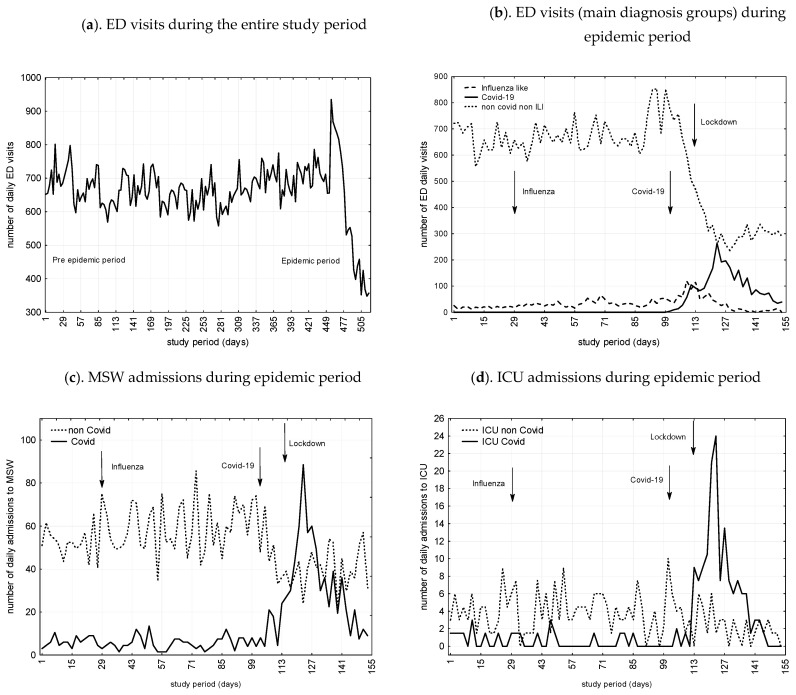
(**a**) ED visits during the entire study period, from 25 November 2018 to 25 November 2019 (pre-epidemic period) and from 26 November 2019 to 26 March 2020 (study period including influenza- and COVID-19-epidemic and lockdown periods); (**b**) ED visits subgroups during Influenza, Covid-19, and lockdown periods (from 26 November 2019 to 26 March 2020); (**c**) MSW admissions during epidemic period (from 26 November 2019 to 26 March 2020); (**d**) ICU admissions during epidemic period(from 26 November 2019 to 26 March 2020). Caption: Admissions are given as a function of 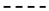 Infuenza like related cases; 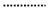 non-COVID related cases; 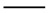 COVID-19 related cases; ED, emergency department; ICU, intensive care unit; MSW, medical/surgical wards.

Figure 2 presents number of ED visits, admissions to MSW and ICU during the study period as a function of COVID-19 and non-COVID related cases. All in all, ED visits and admissions to MSW decreased by 41.7% and 32.6%, while ICU admissions increased by 22.8%. We found significant trends toward decrease during the COVID-19 and lockdown periods for non-COVID admissions to MSW (29.9% and 24.2%) and ICU (71.1% and 13.8%), respectively.

### 3.2. Impact of Influenza, COVID-19 Epidemic, and Lockdown on ED and through-ED Admissions to MSW and ICU

As presented in Table 1, interrupted time series analysis for ED visits, ARIMA models indicate significantly increasing trend after influenza, followed by a significantly decreasing trend after the lockdown. For MSW admissions, we found, first a surge after influenza, then a decline after COVID-19, and finally an even greater decrease during the lockdown period. For ICU admissions, an initial increase after COVID-19 was followed by a decrease after lockdown.

### 3.3. Multifaceted Analysis Model: Predictors of Daily ED Visits and through-ED Admissions to MSW and ICU

ED daily visits were significantly associated in multivariate regression model with the lockdown period and age <65 years, triage level 4/5, arrival mode own resources (adjusted-R2 0.9343). Through ED daily admitted patients to MSW were associated with the number of COVID-19 proved cases, triage levels 1 to 3, and ILI/COVID suspected cases (adjusted-R2 0.4986). Through ED daily admitted patients to ICU were significantly associated with daily triage levels 1 to 3, admitted to MSW and COVID-19 cases proved cases (adjusted-R2 0.4111) (Table 2).

### 3.4. Trends on ED Main Characteristics and Diagnosis Groups

Trends between activity during the seasonal influenza, COVID-19 and lockdown periods and that of the previous year (pre-study period) have been analyzed. We found increasing trends for ED visits (10.6%), and MSW (12.8%) and ICU (4.6%) admissions during the influenza epidemic period. COVID-19 epidemic and lockdown periods showed diminishing trends for ED visits (36.9% and 43.4%). COVID-19 epidemic period exhibited an increased number of MSW (33.9%) and ICU (277%) admissions, mainly related to COVID-19-related admissions (366% and 406%), but non-Covid-admissions were reduced by 29.9% and 71.1%. In contrast, during lockdown period, we observed decreasing trends for MSW (16.8%) and ICU (15.5%), affecting both COVID and non-COVID related cases. They were 2712/101 920 (2.7%) frequent users during the epidemic period. We found significant increasing trends during influenza, COVID-19 and lockdown periods (128.5%, 221% and 188%) (Table 3).

Regarding main diagnostic groups (Table 4) we found a surge during the influenza and COVID-19 epidemic periods for ILI (161% and 225%) and ILI/COVID (244% and 865%), respectively, compared to the pre-study period. During the influenza epidemic period, we found a significant upward trend for acute coronary syndrome (13.7%) and pulmonary embolism/deep vein thrombosis (31.1%). During the COVID-19 epidemic period, while some other diagnostic groups dropped in number, pulmonary embolism/deep vein thrombosis (42.4%) increased. During the lockdown period, while some other diagnostic groups exhibited a downturn comprised between 40.8% and 73.6%, pulmonary embolism/deep vein thrombosis (33.5%) increased. Among non-COVID related conditions, there was a decrease in acute critical conditions as acute coronary syndrome (51.2%), stroke (57.1%), seizures (48.1%), diabetes decompensation/diabetes acidoketosis (47.2%), and surgeries such as appendectomy (57.4%). During the lockdown period, we observed an initial fall followed by a gradual recovery that did not reach usual level for psychiatric conditions, thoracic pain, acute coronary syndrome, atrial fibrillation, hand wounds, head trauma, headaches, abdominal pain, appendicitis, renal colic, urinary tract infection, and acute diabetes decompensation/diabetes acidoketosis. 

## 4. Discussion

Our results indicate that activity of the ED and the hospital, experienced significant variations during the study period. During the influenza epidemic period, we found increasing trends in ED visits, MSW, and ICU admissions (10.6%, 12.8%, and 4.6%, respectively), while for the COVID-19 epidemic period, we observed a decreasing trend in the number of ED visits (36.9%) and also a pattern of growth for MSW and ICU admissions (33.9% and 277.0%, respectively). During lockdown, ED visits and admissions to MSW and ICU declined (49.6%, 16.8%, and 15.5%, respectively). Our results also show that many characteristics of ED visits, mainly age and acuity, and some diagnostic categories, mainly ILI and COVID-19 cases, were associated with changes in activity.

By using time series analysis, we found that the seasonal epidemic influenza period was significantly associated with increased ED visits and admissions to MSW. Our results confirm previously published data indicating increased ED and hospital activity during influenza epidemics [19,20]. During COVID-epidemic, a 50% to 68% reduction in ED visits [7,11] and an increase of hospitalizations in Italy from 22 to 44% were reported [7]. A very recent monocentric study was conducted in Bern, Switzerland, in March 2020. It also showed a decrease in the number of ED visits [12]. In the study of Giamello et al. in Italy [7] and Hautz et al. in Switzerland [12], the periods of study were shorter than in ours. An analysis of a longer period probably enabled us to identify two phases in the period of COVID-19. Our results identify two different situations related to the COVID-19 period. First, during the rapid growth phase of the COVID-19 epidemic, there was a non-significant reduction in the number of ED visits and a notable increase in the number of cases admitted to MSW and to ICU. This trend was followed during the lockdown period, by a reduction in the number of ED visits and admissions to MSW and ICU. We found that the increase in admissions to MSW and ICU was strongly associated with the increase in COVID-19 cases and observed a pronounced decrease of admissions, which was correlated to non-related COVID-19 cases. Other situations may have influenced ICU activity such as patient transfers from other hospitals or admissions of patients initially in MSW who have deteriorated.

In multifaceted regression analysis, we found that the number of ED visits was associated with lockdown period, the fall of ED-visits by young patients with low acuity conditions; through-ED admissions to MSW and to ICU were significantly associated with COVID-19 cases and high acuity patients (adjusted-R2 0.9343, 0.4986, 0.4111, respectively). ED visit characteristics, mainly age and acuity level, and the number of COVID-19 cases made it possible to predict the number of ED visits and admissions to MSW and to ICU. Our results also suggest that syndromic emergency room surveillance of ILI cases, which is strongly related to the number of COVID-19 cases, is of epidemiological interest not only for influenza as previously reported [38] but also for COVID-19.

In addition, we showed for certain diagnostic categories (acute coronary syndrome, stroke and diabetes), traumatic conditions (hip fracture, hand wound, head trauma), pain syndromes (headaches, abdominal pain, renal colic, low back pain), seizures, urinary tract infections and emergency surgical conditions as appendicitis, decreases during the COVID-19 epidemic period, followed by larger decreases during the lockdown period. While in some clinical situations we observed a persistent decrease throughout the lockdown period, in others (psychiatric conditions, thoracic pain, acute coronary syndrome, atrial fibrillation, hand wounds, head trauma, headaches, abdominal pain, renal colic, urinary tract infection, acute diabetes decompensation/diabetes acid ketosis, and appendicitis) we observed a secondary increasing trend but without reaching the expected values. As has been reported, our data indicate that patients may have delayed ED visits [7,8,9,10,11] and access to emergency surgical care [11], even for conditions that might have required hospitalization, including ICU. If for traumatic pathologies we can assume a reduction of their frequency during the lockdown period, this does not seem credible for the other clinical conditions. Hautz et al. suggested various factors that may explain the decline in ED visits. First, the authors suggested a reduction in the need for emergency care due to a reduction in activities that result in trauma problems. Patients with minor complaints may choose to self-treat. Finally, patients who needed to go to the ED may have wished to avoid being infected with COVID-19 in the hospital [12]. In fact, the reasons for reduced patient presentation during new-emerging viral outbreak would appear to be multifactorial [39]. Factors include decreased supply of care; overloading of the health care system; and patients’ fear of the emerging infectious disease, with fears fueled by a certain degree of scientific uncertainty and potentiated by media information management [40,41]. Reduced care for urgent acute situations and decisions to postpone non-essential elective surgeries, chronic disease care, and preventive care [42,43] could compromise the quality of care and result in a loss of opportunity for these patients [44].

There was a significantly increased trend for acute coronary syndromes during seasonal influenza period (13.7%) followed by decrease of more than 50% during the lockdown period. Previous studies found a significant association between recent respiratory infection and acute coronary syndromes [45]. For pulmonary embolism/deep vein thrombosis, we found a significant increase during the seasonal influenza, COVID-19 and lockdown periods (31.1%, 42.4%, and 33.5%). Excess risks in winter for deep vein thrombosis (9%) and pulmonary embolism (22%) [46] were reported, as was an increased risk of thromboembolism among COVID-19 patients [47,48]. Otherwise, increases during the influenza, COVID-19 and lockdown periods (128, 221%, and 188%) were found for frequent users, who have higher prevalence of chronic illness, psychiatric comorbidity, and lower socioeconomic status than non-frequent users [49]. The present results indicate that frequent users need more care during epidemic periods and that EDs were the only place where people could get medical care during lockdown period.

The study was conducted in 4 academic hospitals in the northern region of Paris. This is a region particularly affected by the COVID-19 pandemic. It is a very dense area of Paris where poverty is very present. Hospital activity could be different in other areas of Paris or regions less affected by the COVID-19 pandemic. Nevertheless, we believe that this study provides insights for helping to inform healthcare-related decision making. The present results could enable areas not currently affected by the COVID-19 pandemic to anticipate ED activity and the need for MSW and ICU beds.

As recently reported, non-pharmaceutical interventions [50], notably lockdown periods brought the time-varying reproduction number (R0) near or below 1 and reduced the number of COVID-19 cases and the demand on hospital beds, especially those in ICUs [51]. The present study confirms these results and indicates that in case of new epidemic waves, non-pharmaceutical interventions, mainly lockdown, should be used to reduce COVID-19 cases and their hospital demand. However, our results also indicate the fall demand for hospital care in non-COVID cases during lockdown period may be considered as a secondary side effect with possible severe consequences linked to the delay in diagnosis and in specialized treatment, as recently reported for cancer patients [52]. This study highlights the value of promoting non-COVID patient circuits since this population tends not to come for treatment when necessary. The risk is then to see an initially benign pathology worsen, or even endanger patients due to a delay in care. It is necessary to both preserve certain hospitals and allow them to have an exclusive COVID-19 free activity or dedicate one circuit within the same ED to two separate patient circuits. Similarly, some departments may have a totally COVID-19 free activity and others dedicated to the management of COVID-19 patients. The population should be encouraged to consult the ED when necessary and not give up because of the COVID-19 pandemic.

### Limitations 

The present study is not without limitations. Firstly, the definition of patients suspected of COVID-19 changed as the pandemic evolved. It is possible that few patients who had an infectious syndrome at the onset of the epidemic were infected with COVID-19 but were not detected. This might impact the actual number of COVID-19 cases, as these patients were not reclassified. However, we speculate that the impact was minor, since the first phase of the epidemic certainly had fewer repercussions on ED and hospital activity. Finally, the study was conducted in hospitals where access to care is easy for all patients who can be quickly tested in the ED using a rapid multiplex PCR. Our results could therefore be partially different in hospitals without rapid tests in EDs and in other countries where racial or socio-economic disparities are present. 

## 5. Conclusions

The present study confirms an increase in demand for care at ED, MSW, and ICU during the influenza period and the initial phase of the COVID-19 epidemic and a drop in activity related to non-COVID conditions at ED, MSW, and ICU during the lockdown period. The increase in the number of ILI, COVID-19 and high acuity cases in ED, explains the increase in demand for MSW and ICU beds. Non-pharmaceutical interventions, notably lockdown periods, allowed reducing the number of COVID-19 cases and the demand on hospital beds, especially those in ICUs. On the other hand, and even though the fall in ED activity during the lockdown period primarily involved young patients with low acuity conditions, we observed significant decreases in many complex clinical conditions. Experience of hospital activities during the entire period could help to better prepare for a potential second wave of COVID-19 pandemic and seasonal influenza. Non-pharmaceutical measures should be carried out to reduce the impact of the pandemics. During these periods, patients with other pathologies should not be neglected, and the existence of specific circuits should be created to encourage them to consult in the event of a health problem justifying hospital care. Syndromic surveillance of ILI cases in ED is a tool for monitoring influenza and COVID-19, and it can predict ED activity and the need for MSW and ICU beds. It could help to anticipate and manage future infectious disaster risk in a changing world.

## Figures and Tables

**Table 1:** Time series analysis Interrupted autoregressive integrated moving average (ARIMA) models for influenza epidemic, COVID-19 epidemic, and lockdown periods.

Variables-	Parameter	SE	t (150)	*p*	CI 95%	
Daily visits to ED						
Influenza epidemic period						
Omega	160.8	51.7	3.1	0.002	58.5	263.0
Delta	0.98	0.006	176.4	<0.001	0.97	0.99
COVID-19 epidemic period						
Omega	124.7	110.8	1.1	0.3	−94.3	343.7
Delta	0.6	0.3	1.9	0.06	−0.03	1.3
Lockdown period						
Omega	−160.2	23.8	−6.7	<0.001	<0.001	−113.1
Delta	1.0	0.005	210.6	<0.001	1.01	1.03
Daily through ED admissions to MSW						
Influenza epidemic period						
Omega	6.2	5.4	1.1	0.2	−4.4	16.8
Delta	1.0	0.006	177.6	<0.001	0.99	1.02
COVID-19 epidemic period						
Omega	−31.7	15.4	−2.1	0.04	1.2	62.2
Delta	0.5	0.3	2.0	0.05	−1.1	−0.007
Lockdown period						
Omega	−14.6	7.6	1.9	0.05	−0.4	29.6
Delta	1.0	0.02	48.2	<0.001	0.9	1.0
Daily through ED admissions to ICU						
Influenza epidemic period						
Omega	0.7	0.8	0.9	0.4	−0.8	2.1
Delta	−1.0	0.02	−39.7	<0.001	−1.0	−0.9
COVID-19 epidemic period						
Omega	13.9	2.1	1.8	0.05	−0.3	8.1
Delta	0.8	0.1	−7.5	<0.001	−1.0	−0.6
Lockdown period						
Omega	−13.2	2.3	5.3	<0.001	8.78	17.7
Delta	0.9	0.01	79.52	<0.001	0.9	1.0

Legend: ED, emergency department; ICU, intensive care unit; MSW, medical/surgical wards.

**Table 2:** Multifaceted regression model: predictors of emergency department and hospital activities.

	Beta	SE of Beta	CI 95%	F	P	Adjusted R2
ED daily visits number						
Number of patients aged <65 years	0.2	0.02	0.09–0.2	28.9	<0.001	0.6
Lockdown period	0.3	0.1	0.1–1.0	130.1	<0.001	0.6
Number of triage level 4/5	0.2	0.05	0.1–0.3	26.2	<0.001	0.8
Number of arrival mode by own resources	0.6	0.05	0.5–0.7	179.5	<0.001	0.9
Trough ED daily admitted patients to MSW						
Number of COVID proved cases	0.9	0.1	0.7–1.1	76.9	<0.001	0.3
Number of triage level 1 to 3	0.5	0.07	0.4–0.7	51.5	<0.001	0.4
Number of ILI/COVID suspected cases	−0.7	0.1	−0.9–−0.5	51.7	<0.001	0.5
Trough ED daily admitted patients to ICU						
Number of triage level 1 to 3	0.3	0.07	0.1–0.4	14.3	<0.001	0.3
Number of admitted cases to MSW	0.2	0.07	0.05–0.3	6.8	0.01	0.4
Number of COVID proved cases	0.4	0.07	0.2–0.5	26.2	<0.001	0.4

Legend: ED, emergency department; ICU, intensive care unit; ILI, influenza-like illness; MSW, medical/surgical wards.

**Table 3:** Time series analysis models for influenza epidemic, COVID-19 epidemic and lockdown periods.

Variables-	Influenza Epidemic Period				COVID-19 Epidemic Period				Lockdown Period			
	Trend (%)	CI 95%		*p*	Trend (%)	CI95%		*p*	Trend (%)	CI 95%		*p*
ED and hospital activities												
ED visits	10.6	7.3	13.9	0.01	−36.9	−43.0	−30.7	<0.001	−49.6	−55.7	−43.4	<0.001
Admission to MSW	12.8	9.2	16.4	0.003	33.9	28.1	39.7	<0.001	−16.8	−21.2	−11.4	0.001
non-COVID ED admissions to MSW	-	-	-	-	−29.9	−22.1	−36.3	<0.001	−24.2	−29.3	19.5	<0.001
COVID admissions to MSW	-	-	-	-	366.0	248.0	435.0	<0.001	−68.0	−79.6	−52.1	<0.001
Admissions to ICU	4.6	2.5	6.7	0.05	277.0	260.4	293.6	<0.001	−15.5	−18.2	11.8	−0.02
non-COVID ED admissions to ICU	-	-	-	-	−71.1	−76.3	−64.9	<0.001	−13.8	−18.2	8.4	0.03
COVID admissions to ICU	-	-	-	-	406.0	377.0	486.0	<0.001	−22.4	−26.3	−18.7	0.001
ED visits number as a function of main case characteristics												
Age <65 years	7.6	4.8	10.4	0.008	−18.3	−22.5	−14.1	0.001	−68.3	−75.9	−60.5	<0.001
Male	8.8	5.8	11.8	0.001	9.2	6.2	12.2	0.03	−62.6	−70.3	−54.3	<0.001
Frequent users	128.5	117.2	139.8	<0.001	221	206.1	235.9	<0.001	188.9	174.3	201.7	<0.001
Arrival mode Own resources	22.6	17.8	27.4	<0.001	−28.4	23.1	33.7	<0.001	−52.6	−59.7	−44.8	<0.001
Arrival mode Ambulance	5.9	3.5	8.3	0.005	52.6	45.3	59.9	<0.001	16.6	12.5	20.7	0.001
Triages 1 to 3	16.7	12.6	20.8	0.05	74.4	65.8	83.0	<0.001	−21.6	−26.4	−16.8	0.001
Triages 4 and 5	17.4	13.2	21.6	<0.001	5.2	2.9	7.5	0.09	−63.1	−71.0	−55.1	<0.001

Legend: ED, emergency department; ICU, intensive care unit; MSW, medical/surgical wards.

**Table 4:** Trends in main diagnosis groups during 2019 and 2020 study periods compared to the 2018–2019 period (pre-study period).

Main Diagnosis-	Influenza Epidemic Period				COVID-19 Epidemic Period				Lockdown Period			
	Trend (%)	CI 95%		*p*	Trend (%)	CI 95%		*p*	Trend (%)	CI 95%		*p*
ILI	161.0	148.3	173.7	<0.001	225.0	210.0	240.0	<0.001	−42.1	−48.1	−36.0	<0.001
ILI plus COVID-19 suspected cases	244.0	228.4	259.6	<0.001	865.0	835.6	894.4	<0.001	31.6	26.0	37.2	<0.001
COVID-19 proved cases	125.0	97.0	199.0	<0.001	388.0	296.0	477.0	<0.001	−69.0	33.0	109.0	<0.001
Acute coronary syndrome	13.7	10.0	17.4	0.007	−1.1	−2.0	0.1	1	−51.2	−52.1	−50.0	<0.001
Atrial fibrillation	−26.8	−32.1	−21.1	0.01	−34.2	−39.5	−28.5	0.001	−71.3	−76.6	−65.6	<0.001
Thoracic pain	8.4	5.5	11.3	0.05	−19.2	−23.7	−14.7	0.02	−40.8	−45.3	−36.3	<0.001
Pulmonary embolism/deep vein thrombosis	31.1	25.5	36.7	0.001	42.4	35.9	44.8	<0.001	33.5	27.7	35.9	<0.001
Stroke	1.9	0.5	3.3	0.6	−5.8	−8.2	−3.4	0.09	−57.1	−64.6	−49.4	<0.001
Seizures	3.8	1.9	5.7	0.2	1.1	0.1	2.1	0.7	−48.1	−55.0	−41.2	<0.001
Diabetes	4.2	2.3	6.8	0.3	2.1	1.7	2.8	0.6	−47.2	−53.8	−42.1	0.001
Hip fracture	5.1	2.8	7.4	0.1	1.9	0.5	3.3	0.6	−44.6	−51.2	−38.0	<0.001
Hand wound	6.9	4.3	9.5	0.1	−1.9	−3.4	−0.5	0.3	−51.3	−52.8	−49.9	0.001
Head trauma	-0.8	−1.7	0.5	0.9	−6.6	−7.5	−5.3	0.06	−59.7	−67.4	−52.1	<0.001
Headaches	14.8	11.0	18.6	0.01	5.9	3.5	8.3	0.04	−42.9	−49.2	−36.4	<0.001
Abdominal pain	3.4	1.6	5.2	0.6	2.6	1.0	4.2	0.09	−52.2	−59.5	−45.0	<0.001
Renal colic	12.8	9.2	16.4	0.05	−3.9	−5.9	−1.9	0.2	−54.3	−56.3	−52.3	<0.001
Low back pain	−11.6	−15.1	−8.2	0.04	−22.4	−25.9	−19.0	0.001	−73.6	−81.7	−65.3	<0.001
Appendicitis	14.6	10.8	18.4	0.05	1.9	0.5	3.3	0.6	−57.4	−64.5	−49.6	<0.001
Urinary tract infection	−18.4	−22.8	−14.2	0.01	−31.3	−36.9	−25.7	<0.001	−62.5	−68.1	−56.9	<0.001
Alcoholic intoxication	−7.9	−10.7	−5.1	0.07	−24.8	−27.6	−19.8	<0.001	−73.4	−81.6	−65.0	<0.001
Psychiatric disorders	7.8	5.0	10.6	0.07	−8.4	−11.3	−5.5	0.005	−23.8	−28.5	−18.9	<0.001

Legend: ILI, influenza-like illness.

## Appendix A

Covid-19 cases trend overtime in France (logarithmic scale).
